# Molecular Epidemiological Study of Hand, Foot, and Mouth Disease in a Kindergarten-Based Setting in Bangkok, Thailand

**DOI:** 10.3390/pathogens10050576

**Published:** 2021-05-10

**Authors:** Nipa Thammasonthijarern, Nathamon Kosoltanapiwat, Warisa Nuprasert, Pichamon Sittikul, Pimolpachr Sriburin, Wirichada Pan-ngum, Pannamas Maneekan, Somboon Hataiyusuk, Weerawan Hattasingh, Janjira Thaipadungpanit, Supawat Chatchen

**Affiliations:** 1 Department of Parasitology, Faculty of Veterinary Medicine, Kasetsart University, Bangkok 10900, Thailand; nipa.tha@ku.th; 2 Department of Microbiology and Immunology, Faculty of Tropical Medicine, Mahidol University, Bangkok 10400, Thailand; nathamon.kos@mahidol.ac.th; 3 Department of Tropical Pediatrics, Faculty of Tropical Medicine, Mahidol University, Bangkok 10400, Thailand; warisa_nu@yahoo.com (W.N.); pichamon.sit@mahidol.ac.th (P.S.); pimolpachr.srb@mahidol.ac.th (P.S.); weerawan.hat@mahidol.ac.th (W.H.); 4 Department of Tropical Hygiene, Faculty of Tropical Medicine, Mahidol University, Bangkok 10400, Thailand; wirichada.pan@mahidol.ac.th (W.P.-n.); pannamas.man@mahidol.ac.th (P.M.); 5 Mahidol-Oxford Tropical Medicine Research Unit, Faculty of Tropical Medicine, Mahidol University, Bangkok 10400, Thailand; janjira.tha@mahidol.ac.th; 6 Department of Psychiatry, Faculty of Medicine Siriraj Hospital, Mahidol University, Bangkok 10700, Thailand; somboon.hat@mahidol.ac.th; 7 Department of Clinical Tropical Medicine, Faculty of Tropical Medicine, Mahidol University, Bangkok 10400, Thailand

**Keywords:** HFMD, molecular epidemiology, kindergarten, Thailand

## Abstract

Hand, foot, and mouth disease (HFMD) is a contagious childhood illness and annually affects millions of children aged less than 5 years across the Asia–Pacific region. HFMD transmission mainly occurs through direct contact (person-to-person) and indirect contact with contaminated surfaces and objects. Therefore, public health measures to reduce the spread of HFMD in kindergartens and daycare centers are essential. Based on the guidelines by the Department of Disease Control, a school closure policy for HFMD outbreaks wherein every school in Thailand must close when several HFMD classrooms (more than two cases in each classroom) are encountered within a week, was implemented, although without strong supporting evidence. We therefore conducted a prospective cohort study of children attending five kindergartens during 2019 and 2020. We used molecular genetic techniques to investigate the characteristics of the spreading patterns of HFMD in a school-based setting in Bangkok, Thailand. These analyses identified 22 index cases of HFMD (symptomatic infections) and 25 cases of enterovirus-positive asymptomatic contacts (24 students and one teacher). Enterovirus (EV) A71 was the most common enterovirus detected, and most of the infected persons (8/12) developed symptoms. Other enteroviruses included coxsackieviruses (CVs) A4, CV-A6, CV-A9, and CV-A10 as well as echovirus. The pattern of the spread of HFMD showed that 45% of the subsequent enteroviruses detected in each outbreak possessed the same serotype as the first index case. Moreover, we found a phylogenetic relationship among enteroviruses detected among contact and index cases in the same kindergarten. These findings confirm the benefit of molecular genetic assays to acquire accurate data to support school closure policies designed to control HFMD infections.

## 1. Introduction

Hand, foot, and mouth diseases (HFMDs) are caused by a group of human enteroviruses of the family *Picornaviridae*, a highly diverse group of small, non-enveloped, icosahedral viruses with single positive-strand RNA genomes [1,2]. More than 20 serotypes of enteroviruses cause HFMD, and the most common causative pathogens include enterovirus A71 (EV-A71) and coxsackieviruses A16 (CV-A16) and CV-A6. Different serotypes predominate in different regions such as China, India, and Southeast Asia [3,4,5,6]. HFMD is a highly contagious childhood illness, and its incidence is significantly increasing in the Asia–Pacific region. HFMD annually affects millions of children aged less than 5 years. HFMD is usually mild and self-limiting. HFMD symptoms include fever; vesicular rash on the palms, soles, tongue, or buttocks; sore throat; and ulcers of the lips. Herpangina (HA) is a clinical manifestation of HFMD caused by the same group of enteroviruses, characterized by oral blisters on the roof of the mouth and at the back of the throat without a vesicular rash [7]. Among enteroviruses, EV-A71 infection may cause severe complications, which lead to brainstem encephalitis or pulmonary edema [8]. HFMD outbreaks have killed many children residing in Southeast Asian countries and in China; for instance, regarding the HFMD cases in Mainland China, the morbidity of the survivors increased from 37.6/100,000 in 2008 to 139.6/100,000 in 2013 and peaked in 2012 at 166.8/100,000, with a case fatality rate of 0.03% [9,10,11,12,13,14].

HFMD is mainly transmitted through the fecal–oral route via direct contact (person-to-person) as well as indirect contact with surfaces and objects contaminated with excretions from infected persons. The typical incubation period is 3–5 days, which may last for 2 weeks. Enterovirus infection may be asymptomatic, although contagious. Prolonged shedding of EV-A71 from the throat may last for 4 weeks and for 6–12 weeks in fecal excretions [15,16]. Most cases of HFMD cases involve preschool children aged less than 5 years, although adults may be sporadically infected [14,17]. In Singapore and Hong Kong, the estimated basic reproductive number (R0) ranges from 2.4 to 5.5 for different serotypes [18,19].

Since 2001, the Bureau of Epidemiology, Ministry of Public Health, Thailand (MOPH) has mandated hospital-based surveillance of HFMD [20]. The first large-scale HFMD outbreak occurred in 2012, in which the most prevalent cases were associated with CV-A6, with EV-A71 infections the third most frequent [21,22]. A nationwide HFMD outbreak in 2017 was predominantly caused by EV-A71, which predominated in many provinces of Thailand, as well as by CV-A6 and CV-A16 [23]. Numerous studies show that HFMD is related to environmental and sanitation factors such as high temperatures [24], high rainfall volumes [25,26], antiseptic availability, and personal hygiene [27]. In 2018, the Thai MOPH reported >70,000 cases of HFMD with three deaths [20].

The objectives of this study were to investigate the epidemiology of HFMD and to identify molecular genetic characteristics related to spreading patterns in a real-world setting, specifically in kindergartens and daycare centers in metropolitan Bangkok, Thailand.

## 2. Results

### 2.1. Characteristics of HFMD Cases

During May 2019, we enrolled 515 of 892 students aged 2–6 years and a total of 66 teachers aged 18–60 years. Passive surveillance of children suspected of having HFMD (illness with rashes/blisters on the hands, or feet or mouth ulcers) was performed by teacher-coordinators during the study. Throat and blister swabs from the hand or foot were obtained from index cases during the acute phase, and throat swabs and stools were subsequently collected each week for one month after symptoms disappeared. The rate of HFMD was 22 of 892, or 246.63/10,000 person-years. During the 2019 school year, 14 and 8 subjects from five kindergartens were clinically diagnosed with HFMD or herpangina, respectively. The characteristics of the recruited index cases were as follows: 77% were ill during the rainy season, i.e., from mid-May to mid-October, 50% were males, and the majority attended kindergarten level 1 (approximately 3 years old) (Table 1). Seventeen index cases were treated at hospitals, among which 64% were outpatients, and three patients (14%) were hospitalized because of dehydration. All index cases fully recovered without serious complications. We collected 56 throat swabs and 41 stool samples from 22 index cases. Enterovirus was detected in 18 (82%) index cases, and 5′-UTR real-time RT-PCR (qRT-PCR) was used to analyze 12 HFMD cases and 6 herpangina cases.

### 2.2. Identification of Enteroviruses in Index Cases and Contact Persons

**Figure 1:**
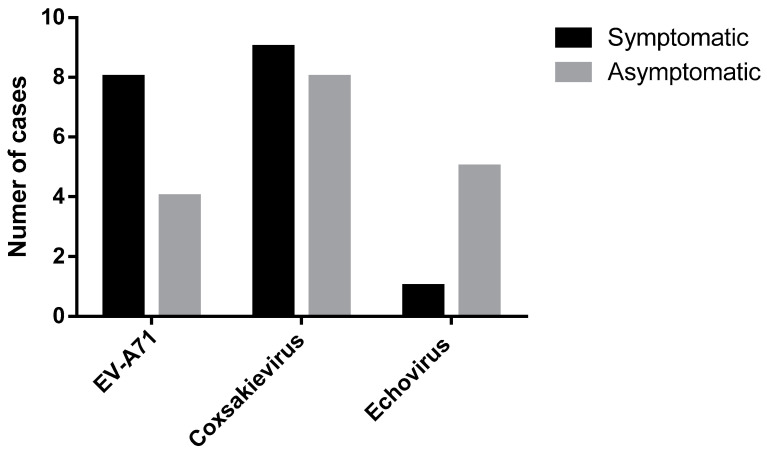
DNA sequence analysis of enteroviruses (EV-A71, CV, and echovirus) of 18 of 22 index cases (black) and 17 of 25 contact persons (gray).

We selected eight contact students and two teachers from the same classroom for enterovirus qRT-PCR analyses of throat swabs as well as five toys related to the index case (169 throat swabs (136 students and 33 teachers) and 53 toy samples from 22 index cases). qRT-PCR assays identified 25 (15%) contact cases (asymptomatic infections) comprising 24 students and 1 teacher. Viral sequences were undetectable in toy samples. Among 18 of 22 (82%) index cases that tested positive for human enterovirus, the 12 HFMD cases comprised 6 (50%) each that tested positive for CV or EV-A71, respectively. Furthermore, among six herpangina-positive cases, 50%, 33%, and 17% were positive for CV, EV-A71, or echovirus, respectively. All causative agents associated with enterovirus-positive index cases (n = 18) were classified using DNA sequencing. In contrast, genotypes of enteroviruses were identified in 17 of 25 (68%) asymptomatic contact cases. The different proportions of enterovirus infections between index cases (symptomatic infections) and contact cases (asymptomatic infections) are shown in Figure 1.

### 2.3. Spreading Pattern of HFMD in Kindergartens

**Figure 2:**
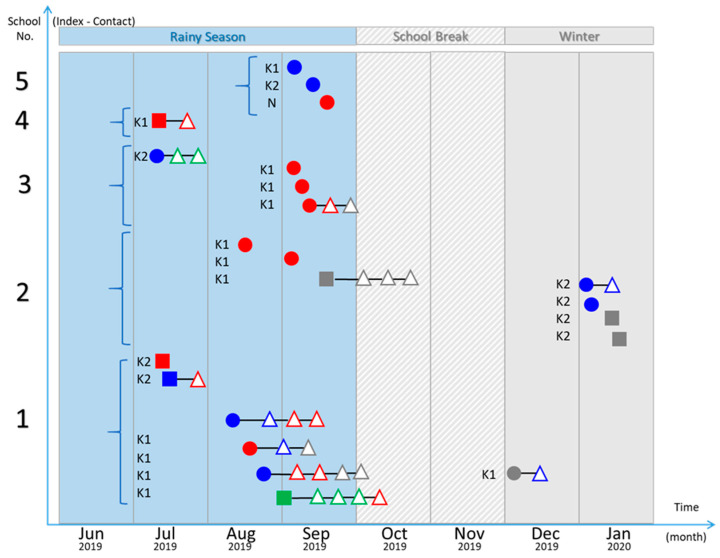
Spreading pattern of HFMD from each kindergarten: Index HFMD cases (filled circles), index cases with herpangina (filled squares), and contact persons (open triangles). DNA sequence analysis of enteroviruses: EV-A71 (blue), CV (red), echovirus (green), and unknown (gray).

Teacher-coordinators from all kindergartens informed 22 families that their children were suspected of having HFMD. Among all index cases, five HFMD events appeared for only one index case in the classroom or grade without causing the subsequent symptomatic infections. Among these events, two from five classrooms showed enterovirus infections in contact students (asymptomatic infections), in which one contact student was infected by the same serotype of enterovirus as the index case. However, the other 17 index cases came from the 5 HFMD outbreaks that occurred in the same classroom or grade among 3 kindergartens. The qRT-PCR analysis detected 22 asymptomatic infections of contact persons from these HFMD outbreaks (on average, 1.29 positive contact persons per index case). Moreover, among the 17 asymptomatic infections classified as enterovirus serotypes, 8 (47%) contact cases harbored the same serotype of enterovirus as the index case of the classroom. Further, among the 22 identified subsequent enterovirus infections (symptomatic and asymptomatic) from the 5 HFMD outbreaks, 10 cases (45%) harbored genotypes related to that of the first index case of each outbreak. EV-A71 was the most prevalent enterovirus, followed by CV-A4 and CV-A6. The spreading patterns of enteroviruses infections from five kindergartens of index cases (symptomatic infections) and contact cases (asymptomatic infections) are shown in Figure 2.

### 2.4. Phylogenetic Analysis of Enteroviruses

**Figure 3:**
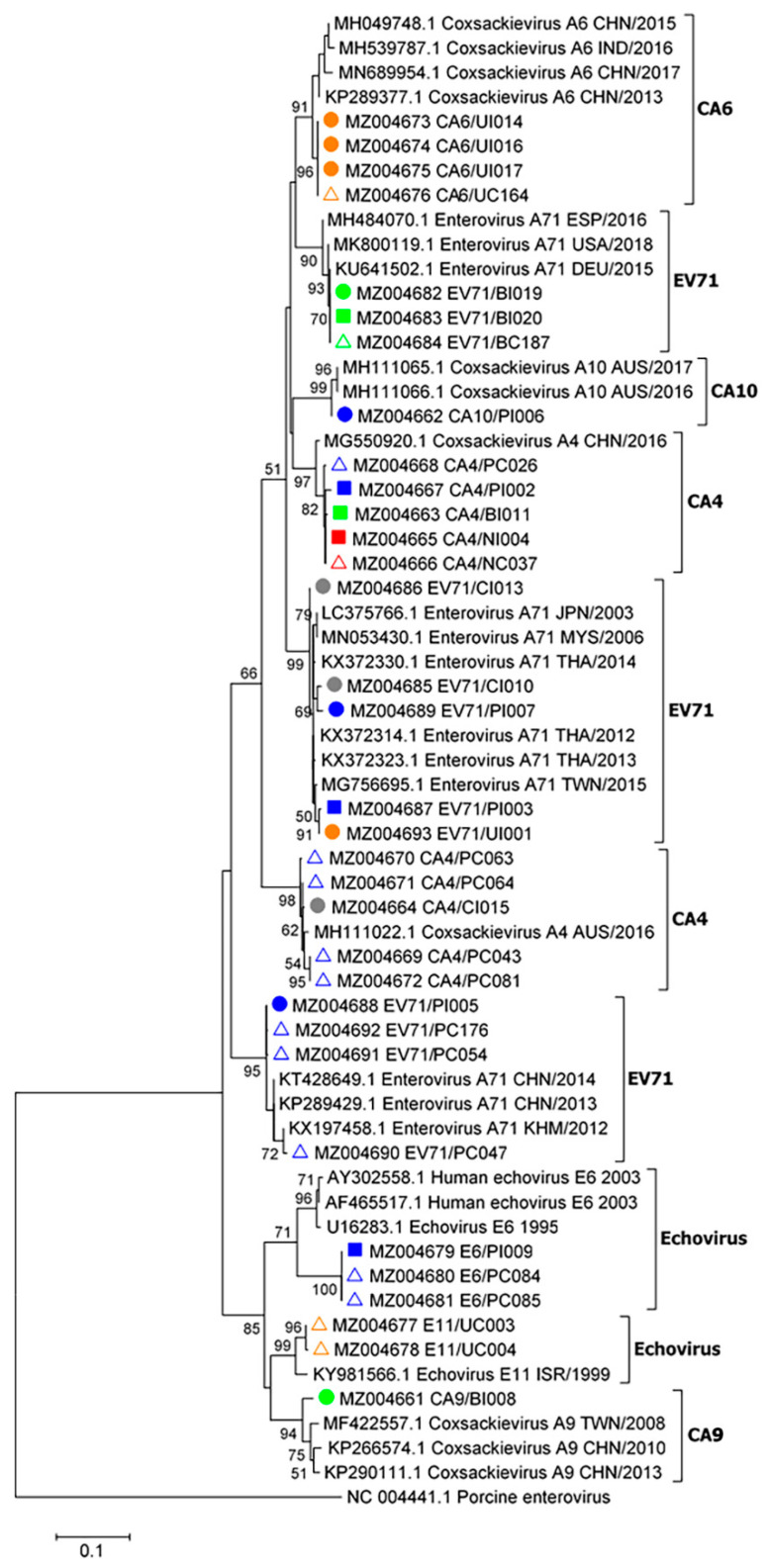
Phylogenetic analysis of partial enteroviral 5′-UTR sequences (410 bp). The tree was constructed using enteroviral sequences obtained from children in five schools and nurseries as follows: school 1 (blue), school 2 (green), school 3 (yellow), school 4 (red), and school 5 (gray). The maximum likelihood method with a bootstrap value of 1000 was applied. Sample names and nucleotide sequence accession numbers are marked with symbols as follows: filled circles, sequences from HFMD cases; filled squares, sequences from cases with herpangina; open triangles, sequences from asymptomatic contact cases. Bootstrap values ≥ 50 are displayed at the nodes. The bar represents nucleotide substitutions per site. A porcine enterovirus sequence served as an out-group.

A phylogenetic tree was inferred using enteroviral partial 5′-UTR sequence data from 17 index cases and 16 asymptomatic contact cases from 5 kindergartens (Figure 3). EV-A71, CV-A4, A6, A9, and A10 as well as echoviruses were found to cause herpangina and HFMD. Our present data suggest that CV-A4 was more likely associated with herpangina, whereas CV-A6 was associated with HFMD. EV-A71 caused herpangina and HFMD. EV-A71, CV-A4, CV-A6, and echoviruses genomes were detected in asymptomatic children. The majority of the enteroviruses shown in the phylogenetic tree are closely related to enterovirus genotypes among each kindergarten. The enteroviral partial 5′-UTR sequences (410 bp) obtained in this study were submitted to GenBank database, National Center for Biotechnology Information (NCBI), National Institutes of Health (NIH). The accession numbers are MZ004661–MZ004693.

## 3. Discussion

We show here that the incidence of HFMD was 246.63/10,000 person-years, which is >20-fold higher than the incidence of HFMD (102.51/100,000 population in 2019) reported by the Thai MOPH [20]. According to the Thai MOPH, the northern region (Chiang Rai, Chiang Mai, and Pha Yao provinces) had the highest incidence of HFMD in Thailand [28,29]. This finding is mainly explained by active surveillance vs. the passive reports of HFMD from all hospitals that are submitted to the Thai MOPH. Moreover, all of the five kindergartens in this study have a history of an HFMD outbreak during 2018.

Asymptomatic enterovirus infections during the HFMD outbreak in the kindergartens identified in the present study averaged 1.29 positive contact persons per index case. Most kindergartens typically assign approximately 25 students per classroom; however, here we investigated up to 8 contact students. Therefore, the true number of positive enterovirus contact persons is likely to be >1.29 per index case. Most asymptomatic infections were detected in contact students, and only one contact teacher was identified.

Numerous studies show that human enteroviruses classified from hospitalized patients with HFMD include different proportions of serotypes [24,30,31]. For example, EV-A71, CV-A16, and CV-A6 are the major causes of HFMD in Thailand [24,30,31]. Here we found that 50% of the detected pathogens from index cases were coxsackieviruses (CV-A4, A6, A9, and A10) and 44% were EV-A71. Thus, EV-A71 was dominant in the enterovirus group, followed by CV. However, different proportions of serotypes of the coxsackievirus group are explained by many factors such as asymptomatic infections [32,33].

HFMD is transmitted via direct and indirect contact with surfaces and objects contaminated by excretions from infected persons. Risk factors for HFMD in kindergartens and daycare centers are mainly environmental, sanitary, and hygienic [34,35]. A mathematic model of HFMD in Bangkok indicates that direct transmission from asymptomatic persons is an important factor [36]. The present study reveals that the possible source of infection was through contact with enterovirus-positive asymptomatic persons in the classroom. Moreover, after one week of symptomatic HFMD, the now asymptomatic index patients usually recovered and returned to school where they shed enterovirus as an asymptomatic contact.

According to the pattern of spread of HFMD in each kindergarten, 45% of the subsequent enteroviruses in each outbreak were the same serotype as the first index case. In contrast, 55% of enteroviruses were unrelated and may have originated from an outside source. Therefore, public health measures such as hand washing and personal hygiene are essential for reducing the spread of HFMD in kindergartens [37]. Nevertheless, a school closure policy based only on the number of HFMD cases, without testing all students for enterovirus infection, may not sufficiently address the actual status of an outbreak and may cause many students to lose the chance to study in a normal school setting [38].

Here we show the close phylogenetic relationships among enteroviruses through our analyses of contact and index cases in the same kindergarten. These findings suggest that subsequent enteroviruses with the same serotype as the first index case were closely related to the enterovirus that spread through the kindergarten. However, these findings do not exclude the possibility of subsequent HFMD cases caused by other enterovirus serotypes present outside of the schools. Therefore, molecular genetic identification of common enterovirus serotypes that cause HFMD in each child may provide information sufficient to prevent an outbreak of HFMD in kindergartens and will likely serve to comprehensively inform school closure policies.

## 4. Materials and Methods

### 4.1. Ethics Statement

All data were confidentially and anonymously handled. The Ethics Committee of the Faculty of Tropical Medicine, Mahidol University reviewed and approved the study protocol (No. TMEC 19-017).

### 4.2. Study Site

**Figure 4:**
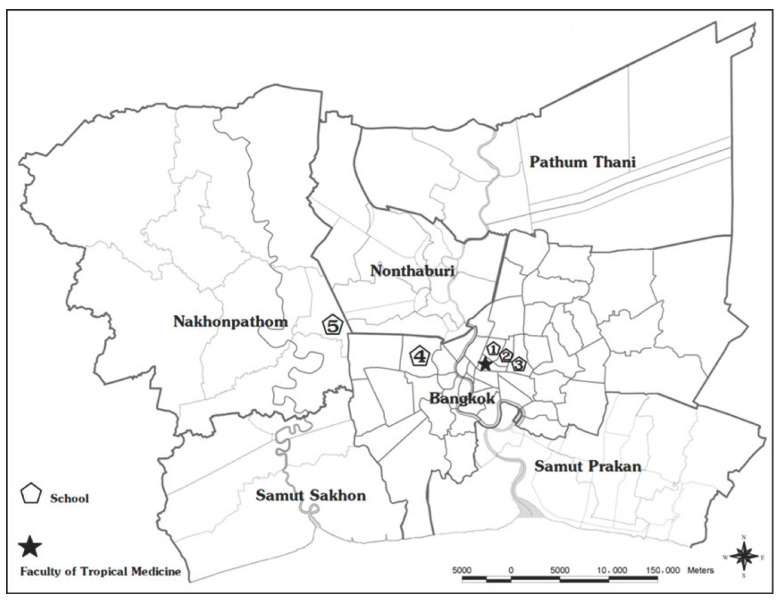
Participating kindergartens in the Bangkok metropolitan region (Bangkok and Nakhonpathom Provinces), Thailand, 2019–2020. Pentagons indicate the locations of five kindergartens, and the star indicates the location of the Faculty of Tropical Medicine, Mahidol University.

The study was performed in the Bangkok metropolitan region (Bangkok and Nakhonpathom Provinces), Thailand. Bangkok is the capital and the most populous city of Thailand, with an approximate population of 10 million and >500 kindergartens. Five kindergartens in this study had a history of an HFMD outbreak during 2018 and are located within 30 km of the Faculty of Tropical Medicine, Mahidol University (Figure 4). There are four government-operated kindergartens and one private kindergarten, with student populations ranging from 100 to 400 students.

### 4.3. Study Design and Specimen Collection

This was a prospective cohort study of children attending 5 kindergartens. Kindergartens were selected according to their desire to participate. Following school-based informational meetings with parents, informed parental consent was obtained from potential participants. Enrollment criteria were as follows: healthy children between the ages of 2 and 6 years (nursery and kindergarten, 1 and 3 years) at the time of enrollment and healthy teachers aged 18–60 years who were employed by one of the study’s kindergartens. The study was conducted to estimate the incidence of HFMD, which was calculated according to the number of new cases divided by the total number of children aged <6 years over 1 year. Informed consent was obtained from all enrolled subjects. During the study (May 2019–April 2020), active surveillance of children suspected of having HFMD (illness with rashes/blisters on the hands and feet, or mouth ulcers) was informed by teacher-coordinators during the school term.

**Figure 5:**
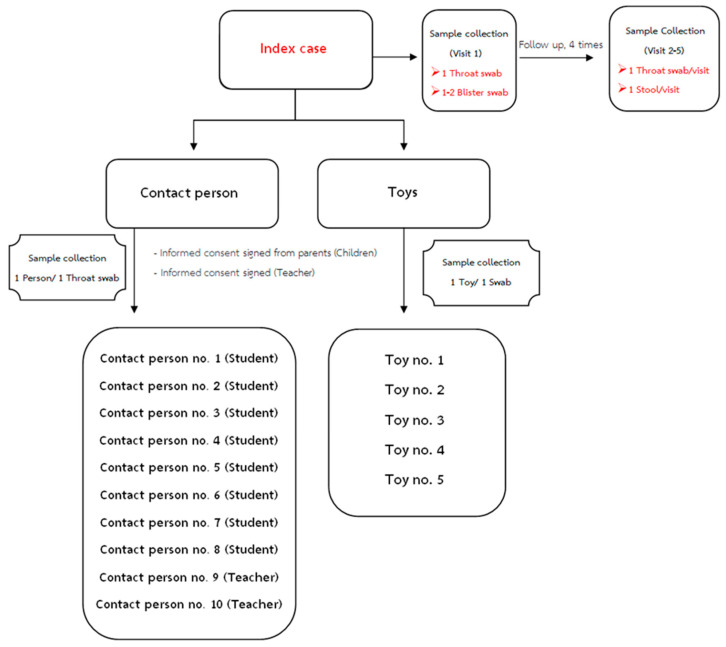
Specimens collected from children with the clinical diagnosis of HFMD or herpangina (eight contact students and two teachers in the same classroom). Five toys were collected.

Specimens from children with the clinical diagnosis of HFMD or herpangina were obtained. For index cases, an acute-phase throat swab and blister swabs of the hand or foot were obtained, and recovery-phase throat swabs and stool samples were collected weekly for one month. Further, clinical data were recorded on CRF during acute illness until recovery. At the kindergarten, throat swabs were collected from up to eight contact students and two teachers in the same classroom, and five toys related to the HFMD cases in the classroom were obtained (Figure 5). Throat swabs were collected 24–72 h after clinical diagnosis, stored in viral transport media or universal transport media (UTM) (COPAN, USA) and sent to our laboratory for molecular diagnosis. Throat swabs were collected with the aid of a tongue depressor, by carefully swabbing the lateral and posterior pharynx. Specimens included 225 throat swabs, 53 toy swabs, and 41 stool samples. All swabs and stool samples were stored at −80 °C.

### 4.4. Sample Preparation and RNA Extraction

Throat swabs collected in 3 mL of UTM (BD, Becton, MD) and supernatants were prepared by vortexing and centrifugation of samples at 3000 rpm for 5 min. Stool samples were processed using the standard operating procedure as follows: 2 g of each stool, 10 glass beads, and 5 mL of UTM were added, vortexed for 3–5 min, and then centrifuged at 3000 rpm for 30 min, after which 4.5 mL of supernatant was mixed with 0.5 mL of PBS containing penicillin/streptomycin. Total RNA was extracted using a QIAamp Viral RNA Mini Kit (Qiagen, Hilden, Germany) according to the manufacturer’s instructions. The RNA was suspended in a final volume of 50 μL of elution buffer and stored at −80 °C.

### 4.5. Detection of Enteroviral 5′-UTR Sequences Using qRT-PCR

Total RNA was subjected to one-step real-time RT-PCR with primers and a probe specific for enteroviral 5′-UTR (Table 2). The qRT-PCR reactions were performed using TaqMan Fast Virus 1-Step Master Mix (Applied Biosystems, CA, USA) utilizing the primers and the TaqMan probe designed for broad detection of enteroviruses including EV-A71, coxsackievirus, and echoviruses. Pan-enterovirus screening primers were EQ1 (Forward) and EQ2 (Reverse), and the specific probe EPmod was labeled at its 5′-terminus with the FAM reporter and at its 3′-terminus with the BHQ1 quencher [39]. Real-time reverse transcriptase PCR assays were performed using the Touch Real-Time Reverse Transcriptase PCR Detection System, CFX96 (Bio-Rad, CA, USA) as follows: 10-min reverse transcription at 50 °C, 20 s denaturation at 95 °C, and 40 cycles at 95 °C for 5 s and 55 °C for 30 s + plate read. Positive control (EV-A71 and CV-A6 virus cultures) and negative control (nuclease-free water) were included in each reaction mixture.

### 4.6. Amplification of Enteroviral 5′-UTR Sequences Using RT-PCR, DNA Sequencing, and Phylogenetic Analysis

Nested RT-PCR was performed to amplify a region of the enteroviral 5′-UTR for DNA sequencing. First-step RT-PCR was performed in 20 µL reaction mixture containing 10 µL of 2× PCR buffer, 1 µL of 100 mM DTT, 3.2 µL of nuclease-free water, 0.8 µL of SuperScript III One-Step System with Platinum Taq High Fidelity (Invitrogen, USA), 0.5 µL of each forward primer (10 µM) (UniEV_5UTR-F76) and reverse primer (UniEv_5UTR-F644), and 5 µL of template. The primer sequences are shown in Table 2. RT-PCR amplification was performed as follows: reverse transcription for 30 min at 50 °C and initial denaturation for 5 min at 94 °C, 40 cycles of denaturation at 94 °C for 15 s, annealing at 50 °C for 30 s, and extension at 72 °C for 1 min. The resulting 568 bp PCR product was analyzed using a 1.5% agarose gel. Nested PCR was performed in a 20 μL reaction containing 10 µL of 2× nested PCR reaction mix (Vivantis, Malaysia), 6.8 µL of nuclease-free water, 0.2 µL of Taq DNA polymerase (Vivantis, Malaysia), 0.5 µL of nested primers (10 µM each) (UniEV_5UTR-F172 and UniEv_5UTR-F610, Table 2), and 2 µL of DNA template from the first-step RT-PCR (diluted 1:50). The thermocycling conditions were as follows: initial denaturation for 2 min at 94 °C, 30 cycles of denaturation at 94 °C for 30 s, annealing at 50 °C for 30 s, and extension at 72 °C for 1 min. Final concentrations of RT-PCR and nested PCR components are listed in Table 3. Nested PCR products of the expected size (438 bp), which were purified from 1.5% agarose gel using a NucleoSpin Gel and PCR Clean up Kit (Macherey-Nagel, Germany), were sent for DNA sequencing (Macrogen, Seoul, South Korea) using both inner primers. The sequencing chromatograms were inspected and processed using BioEdit, version 7.2.5. Nucleotide sequences were used as BLAST queries to search the NCBI database and aligned with reference sequences using the ClustalW component of BioEdit. Phylogenetic trees were constructed using Molecular Evolutionary Genetics Analysis (MEGA, version 7.0.26) software [40]. The maximum likelihood method with the Kimura 2-parameter model was applied according to a phylogenetic model analysis [40]. Bootstrap resampling analysis of 1000 replicates was performed.

## 5. Conclusions

HFMD is a common illness in children below 5 years of age. Certain public health measures reduce the spread of HFMD in kindergartens, and improvements in school closure policies are clearly required. Here we conducted a prospective school-based setting study of five kindergartens during 2019 and 2020 in Bangkok, Thailand. We detected 22 index cases of HFMD and 25 enterovirus-positive contact persons. Asymptomatic infections in kindergartens were identified, with an average of 1.29 positive contact persons per index case. EV-A71 was the most common enterovirus, followed by other enteroviruses such as CV-A4 and CV-A6. The spreading pattern of HFMD suggests that 50% of subsequent enteroviruses detected represented the same serotype and were thus closely related to the first index case. The actual incidence of HFMD is significantly higher than that reported officially. In this study, it is noted that enteroviruses circulating in schools predominantly cause asymptomatic infections. Furthermore, the diversity in enteroviruses may have caused the pattern in which HFMD spreads to be more complex. Moreover, the present study reveals the importance of using molecular genetic assays to detect HFMD. Such analyses potentially provide the sufficiently robust evidence required to support future formulation of school closure policies to control HFMD outbreaks.

## Figures and Tables

**Table 1:** Characteristics of HFMD index cases.

School	Number	Sex		Grade			
		M	F	Nursery	K 1	K 2	K 3
1	7	4	3	0	5	2	0
2	7	3	4	0	3	4	0
3	4	2	2	0	3	1	0
4	1	0	1	0	1	0	0
5	3	2	1	1	1	1	0
Total	22	11	11	1	13	8	0

**Table 2:** PCR primers and probe.

Primer Name	Sequence (5′–3′)
EQ1 (Forward)	5′-ACATGGTGTGAAGAGTCTATTGAGCT-3′
EQ2 (Reverse)	5′-CCAAAGTAGTCGGTTCCGC-3′
EPmod probe (FAM)	5′-ATTAGCCGCATTCAGGGGCCGGA-3′
UniEV_5UTR-F76 (Forward)	5′-GDAYCTTTGTGCGCCTGTT-3′
UniEv_5UTR-F644 (Reverse)	5′-GCCAATCCAATAGCTATATGG-3′
UniEV_5UTR-F172 (Forward)	5′-GRTCAAGCACTTCTGTHTCC -3′
UniEv_5UTR-F610 (Reverse)	5′-ATTGTCACCATAAGCAGCCA-3′

**Table 3:** RT-PCR and nested PCR reaction components.

RT-PCR		Nested PCR	
Component	Final Concentration	Component	Final Concentration
2× reaction mix	1×	2× PCR buffer (Vivantis)	1×
Forward primer	0.25 µM	Forward primer	0.25 µM
Reverse primer	0.25 µM	Reverse primer	0.25 µM
SS III RT/mix platinum *Taq*	0.8 µL	Vivantis *Taq* DNA polymerase	0.2 µL
100 mM DTT	5 mM	nuclease-free water	6.8 µL
nuclease-free water	3.2 µL	1:50 diluted template	2 µL
RNA template	5 µL		

## Data Availability

In this section, please provide details regarding where data supporting reported results can be found, including links to publicly archived datasets analyzed or generated during the study. Please refer to suggested Data Availability Statements in section “MDPI Research Data Policies” at https://www.mdpi.com/ethics. You might choose to exclude this statement if the study did not report any data.
